# Stress Elicits Contrasting Effects on the Structure and Number of Astrocytes in the Amygdala versus Hippocampus

**DOI:** 10.1523/ENEURO.0338-18.2019

**Published:** 2019-02-12

**Authors:** Saptarnab Naskar, Sumantra Chattarji

**Affiliations:** 1 National Centre for Biological Sciences, Bangalore 560065, India; 2 Centre for Brain Development and Repair, Institute for Stem Cell Biology and Regenerative Medicine, Bangalore 560065, India; 3 Centre for Discovery Brain Sciences, Deanery of Biomedical Sciences, University of Edinburgh, Hugh Robson Building, Edinburgh EH89XD, United Kingdom

**Keywords:** basolateral amygdala, chronic immobilization stress, glia, hippocampal area CA3, plasticity, pyramidal neurons

## Abstract

Stress causes divergent patterns of structural and physiological plasticity in the hippocampus versus amygdala. However, a majority of earlier studies focused primarily on neurons. Despite growing evidence for the importance of glia in health and disease, relatively little is known about how stress affects astrocytes. Further, previous work focused on hippocampal astrocytes. Hence, we examined the impact of chronic immobilization stress (2 h/d, 10 d), on the number and structure of astrocytes in the rat hippocampus and amygdala. We observed a reduction in the number of glial fibrillary acidic protein (GFAP)-positive astrocytes in the basal amygdala (BA), 1 d after the end of 10 d of chronic stress. Detailed morphometric analysis of individual dye-filled astrocytes also revealed a decrease in the neuropil volume occupied by these astrocytes in the BA, alongside a reduction in the volume fraction of fine astrocytic protrusions rather than larger dendrite-like processes. By contrast, the same chronic stress had no effect on the number or morphology of astrocytes in hippocampal area CA3. We also confirmed previous reports that chronic stress triggers dendritic hypertrophy in dye-filled BA principal neurons that were located adjacent to astrocytes that had undergone atrophy. Thus, building on earlier evidence for contrasting patterns of stress-induced plasticity in neurons across brain areas, our findings offer new evidence that the same stress can also elicit divergent morphological effects in astrocytes in the hippocampus versus the amygdala.

## Significance Statement

Despite accumulating evidence on the role of astrocytes in health and disease, relatively little is known about the effects of stress on these cells. We report here, for the first time, that the divergent effects of chronic immobilization stress on the morphological properties of neurons in hippocampus and amygdala also extend to astrocytes in the rat brain. Chronic stress reduces the number of astrocytes in the amygdala, without affecting them in the hippocampus. Strikingly, the same stress elicits divergent morphological effects, neuronal hypertrophy versus atrophy of astrocytes, in the amygdala. These findings underscore the need to further examine non-cell autonomous, astroglial-mediated effects elicited by stress, and how these vary across brain regions.

## Introduction

Repeated stress elicits divergent effects on two brain regions, the amygdala and hippocampus that are key regulators of the stress response. Studies using different forms of chronic stress have shown that pyramidal neurons in the hippocampus undergo dendritic atrophy along with a reduction in spine density (Vyas et al., 2002; Pawlak et al., 2005). On the other hand, principal neurons in the basolateral amygdala (BLA) undergo dendritic hypertrophy alongside an increase in dendritic spine density (Vyas et al., 2002; Mitra et al., 2005). Physiological and molecular measures of synaptic plasticity also exhibit contrasting features in these two brain areas (McEwen, 1999; Reagan et al., 2008; Chattarji et al., 2015). Although these earlier studies have given rise to a useful framework for investigating a range of cellular effects of stress in the brain, much of these analyses were focused on neurons. Relatively little is known about how stress affects non-neuronal cells, such as glia, which by some estimates constitute around 50% of the total cell population in the human brain (Herculano-Houzel, 2014).

Among the glial cell population in the brain, astrocytes are known to play a significant role in synaptic transmission and plasticity (Volterra and Meldolesi, 2005). Astrocytes express the glutamate transporter 1 (GLT-1) and glutamate aspartate transporter (GLAST), which form the major glutamate reuptake machinery (Anderson and Swanson, 2000). Further, rodent astrocytes, similar to neurons, express receptors for the primary stress hormone corticosterone, namely the glucocorticoid receptors (GRs) and mineralocorticoid receptors (MRs) (Wang et al., 2014). As elevated levels of systemic corticosterone and extracellular glutamate are some the early manifestations of stress-induced changes, astrocytes are strategically positioned to respond to the physiological consequences of stress at the level of neurons and their connections.

Growing evidence from recent studies point to the important role played by astrocytes in the central nervous system in both health and disease. However, only a handful of studies have focused on the impact of stress on these cells. In one pioneering study, chronic psychosocial stress was shown to cause a reduction in astrocyte numbers in the hippocampus of tree shrews (Czéh et al., 2006). However, there is no comparable analysis in animal models of the effects of stress on astrocytes in the amygdala. A clinical study, on the other hand, reported a reduction in astrocyte numbers in the amygdala of patients suffering from major depressive disorder (Altshuler et al., 2010). This raises questions about the brain region-specific effects of repeated stress on astrocytes. For instance, does repeated stress reduce the number of astrocytes in the amygdala, as has been observed in the hippocampus? As mentioned earlier, accumulating evidence points to contrasting morphological effects of the same chronic stress on principal neurons in the amygdala versus hippocampus. This gives rise to a different possibility, the same chronic stress could have the opposite effect on the number of astrocytes in these two brain areas. Further, astrocytes exhibit a highly ramified, spongiform morphology with non-overlapping physical domains with neighboring astrocytes (Bushong et al., 2002). In addition to the effects of stress on the number of astrocytes, does it also affect the morphological properties of astrocytes? Would these effects, if any, vary between the hippocampus and amygdala? Hence, the present study combines morphometric and histological analyses to address these questions using a well-characterized rat model of chronic immobilization stress (CIS; Vyas et al., 2002; Mitra et al., 2005; Ghosh et al., 2013; Suvrathan et al., 2014; Rahman et al., 2016).

## Materials and Methods

### Experimental animals

Male Sprague Dawley rats were housed in a 14/10 h light/dark schedule (lights on at 8 A.M.) with *ad libitum* access to food and water at the National Centre for Biological Sciences, Bangalore, India. All animal care and experimentation procedures were approved by the Institutional Animal Ethics Committee National Centre for Biological Sciences and Committee for the Purpose of Control and Supervision of Experiments on Animals, Government of India.

### Stress protocol

**Figure 1.**
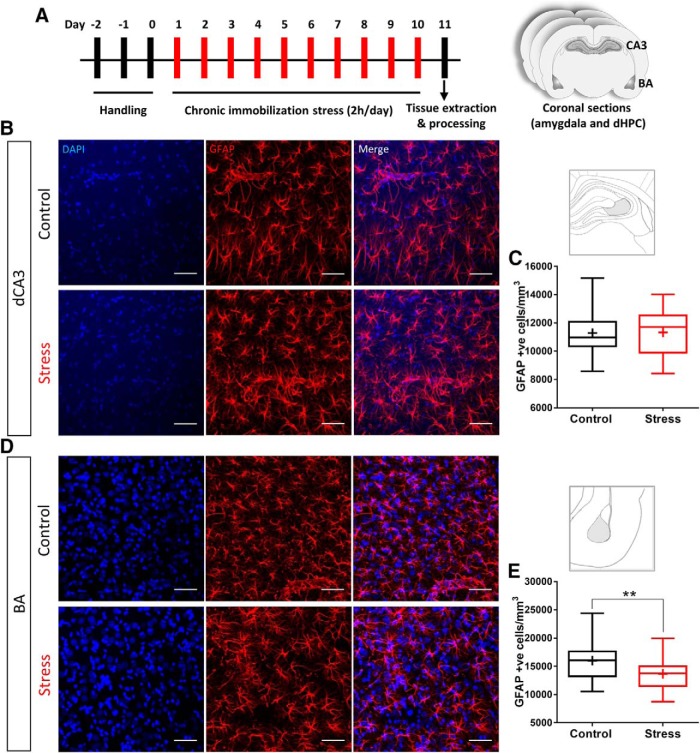
CIS causes a reduction in GFAP-positive astrocytes in BA but not in dCA3. ***A***, Schematic for experimental protocol; 60-d old male Sprague Dawley rats are subjected to CIS protocol. Each bar represents a day with 1 episode of 2-h immobilization for consecutive 10 d. Coronal sections containing dorsal hippocampus (dHPC) and the basolateral amygdala are obtained after transcardial perfusion with 4% PFA. The sections are then subjected to immunofluorescence protocol for GFAP or intracellular dye fills. ***B***, Representative image of GFAP-positive cells in dCA3; top, control; bottom, stress. ***C***, No significant change was observed in average GFAP-positive astrocyte numbers in dCA3 with stress (control: *n* = 46 fields of dCA3 from five animals; stress: *n* = 55 fields of dCA3 from six animals). ***D***, Representative image of GFAP-positive cells in BA; top, control; bottom, stress. ***E***, Average of GFAP-positive astrocyte numbers in BA shows a significant reduction with stress (Kolmogorov–Smirnov test, **p < 0.01; control: *n* = 32 fields of BA from five animals; stress: *n* = 34 fields of BA from six animals). Scale bar: 50 µm.

For CIS, rats were subjected to 2 h of complete immobilization (Mitra et al., 2005) between 10 A.M. and 12 P.M. in plastic immobilization bags for 10 consecutive days (Fig. 1*A*). Rats had no access to food or water during this 2-h period. However, they had access to air through a sufficiently large opening at the tip of the immobilization bags adjacent to the rat’s nose. After stress, animals were returned to home cages. Control animals were not subjected to any type of stress and were housed in a different room.

### Tissue preparation

Twenty-four hours after completion of the 10-d CIS protocol, the animals were anesthetized with an overdose of ketamine and xylazine and perfused transcardially with 50 ml of 0.1 M phosphate buffer (PB) followed by 100 ml of ice-cold fixative solution containing 4% 0.1 M PBS paraformaldehyde (pH 7.4). The brains were then gently removed out from the skull. After post fixation for 3 h in the above-mentioned fixative, coronal sections of the brain containing the amygdala and dorsal hippocampus were obtained using a vibratome (VT1200, Leica) and either stored in 0.1 M PB containing 0.1% sodium azide for immunofluorescence labeling (section thickness: 60 µm) or 0.1 M PB for intracellular fills (section thickness: 100 µm).

### Immunohistochemical labeling

Tissue slices were washed three times for 10 min each in 0.1 M PB containing 0.5% Triton X-100 (TX). Sections were blocked in PB containing 2% normal goat serum (NGS), 3% bovine serum albumin, and 0.3% TX for 3 h at room temperature. The slices were then incubated for 48 h (4°C) in mouse monoclonal anti-glial fibrillary acidic protein (GFAP) antibody (MAB360, Millipore) diluted 1:10,000 in working buffer (PB containing, 2% NGS, and 0.3% TX). The slices were then washed three times for 10 min each in working buffer and then placed in working buffer (25°C) containing 1:500 goat anti-mouse IgG conjugated to Alexa Fluor 633 (Thermo Fisher Scientific). After 3 h, the sections were then incubated for 15 min (25°C) in working buffer containing 1:2000 DAPI (Sigma). The slices were washed three times for 10 min each in PB containing 0.5% TX and then mounted in ProlongGold antifade reagent (Thermo Fisher Scientific).

### Intracellular fills of astrocytes in fixed tissue

**Figure 2.**
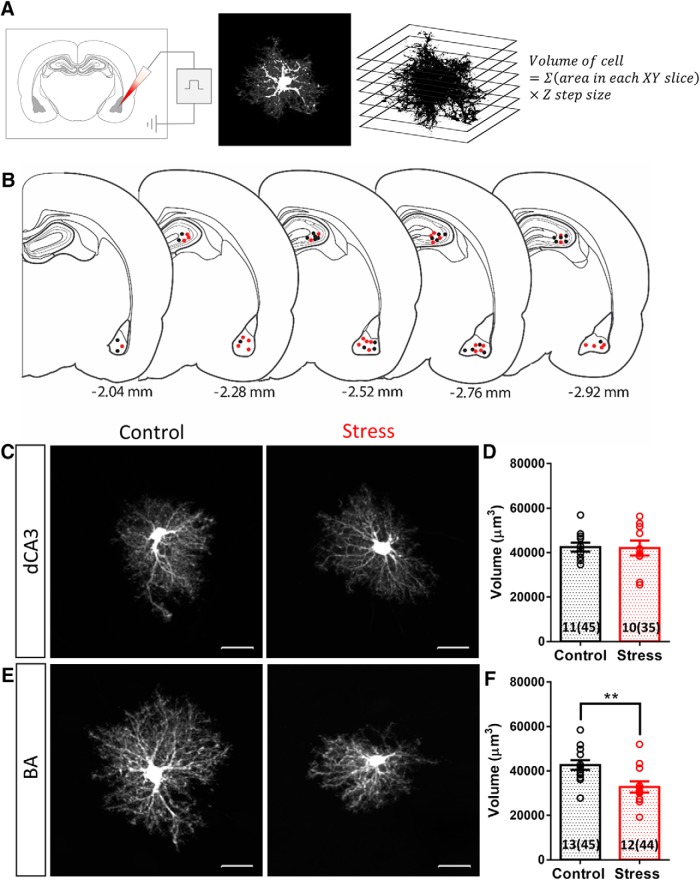
Chronic stress causes a reduction in the neuropil volume occupied by astrocytes in BA but not in dCA3. ***A***, Schematic of experimental protocol: individual astrocytes were filled with Alexa Fluor 568 dye by sharp electrode iontophoresis. The confocal image of a filled cell was converted to binary format and the volume occupied by the cell was calculated by the equation mentioned. ***B***, Location of dye-filled astrocytes on coronal brain sections in reference to the bregma. The dots represent individual cell locations from one batch of animals. Controls: black; CIS: red. ***C***, Representative image of dye-filled astrocytes in dCA3: left, control; right, stress. ***D***, No significant difference was observed in the neuropil volume occupied by dCA3 astrocytes with stress (control: *N* = 11 animals, *n* = 45 cells; stress: *N* = 10 animals, *n* = 35 cells). ***E***, Representative image of dye-filled astrocytes in BA: left, control; right, stress. ***F***, Average neuropil volume occupied by BA astrocytes shows a significant reduction with stress (Student's unpaired *t*-test, **p < 0.01; control: *N* = 13 animals, *n* = 45 cells; stress: *N* = 12 animals, *n* = 44 cells). Bar insert, Number of animals and number of cells (in parentheses). Scale bar: 20 µm.

The method for filling cells in fixed tissue slices was adapted from previously reported protocol (Bushong et al., 2002). The sections, stored in ice-cold PB, were used within 48 h. The sections were placed in cold PB and viewed with an infrared differential interference contrast (DIC)/epifluorescent microscope (SliceScope, Scientifica), using a 40× water immersion objective (Olympus). Sharp glass micropipettes were pulled on a flaming brown pipette puller (P-97, Sutter Instruments) using thin walled glass capillaries with filament (GC150TF, Harvard Apparatus: outer diameter of 1.5 mm and inner diameter of 1.17 mm; resistance ranged between 60 and 100 MΩ) and backfilled with 10 mM Alexa Fluor 568 hydrazide, sodium salt solution (Thermo Fisher Scientific). The astrocytes and principal neurons were identified by the distinctive size and shape of their soma. The somata were impaled, and the dye was injected into the cells by applying a 0.5-s negative current pulse (1 Hz) using Master8 (A.M.P.I. Systems) until the processes were completely filled (∼5 min for astrocytes and ∼15 min for neurons; Fig. 2*A*). After several cells were filled in a tissue section, the section was placed in cold 4% paraformaldehyde-PB for 30 min followed by DAPI (Sigma) staining for 15 min. The sections were then mounted in ProlongGold antifade reagent (Thermo Fisher Scientific).

### Imaging

All image acquisition was done using confocal laser scanning microscopy on Olympus Fluoview 1000 (Olympus) after at least 24 h of mounting. For GFAP immunofluorescence, the regions of interest, i.e., basal amygdala (BA) and area CA3 of the dorsal hippocampus (dCA3) were visualized under 20× objective and confocal stacks spanning the section thickness was acquired at a Z-step size of 1 µm. The imaging conditions including PMT voltage and laser power were kept constant for all the GFAP imaging. For intracellular fills, the filled cells were visualized under a 40× oil immersion objective and confocal stacks spanning the cell was acquired at a Z-step size of 0.5 µm. The Z-step size was according to the Nyquist sampling criteria based on the axial resolution of the imaging system. The PMT voltage was adjusted for each cell such that the soma was completely saturated. This was done to have a uniform representation of all cells irrespective of minor variations in the amount of dye inside a cell.

### Image visualization and analysis

**Figure 3.**
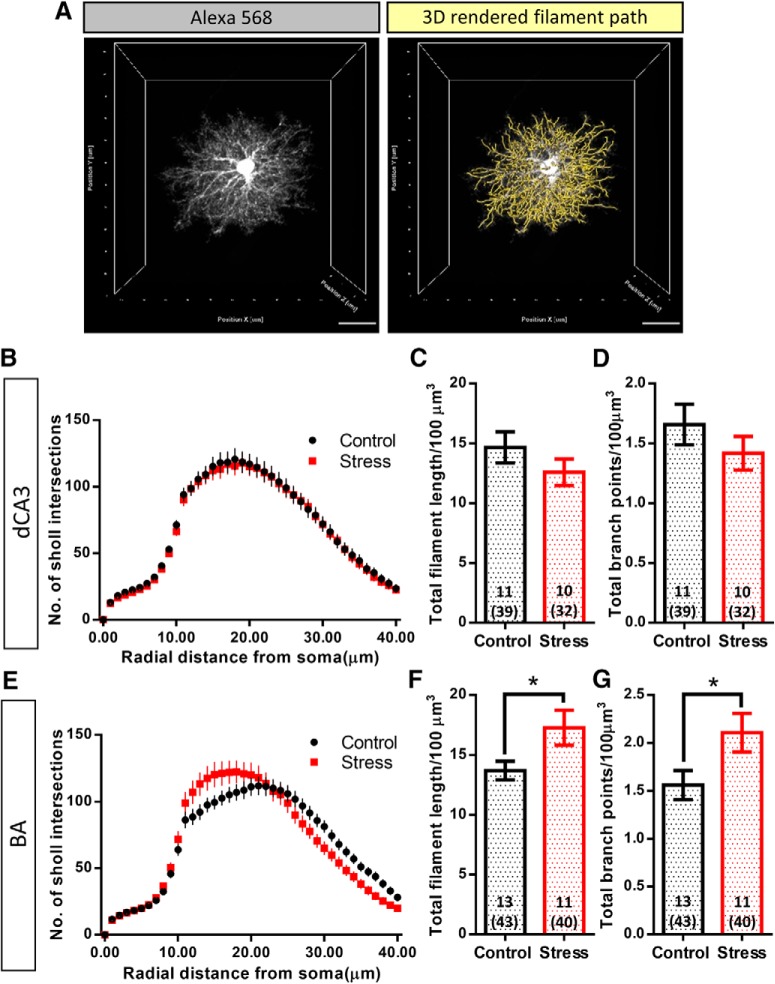
Stress does not affect the branching complexity of large dendrite-like processes of astrocytes but increases their packing density in BA astrocytes. ***A***, left, A representative image of Alexa Fluor 568-filled astrocyte. Right, The 3D filament path skeleton (yellow lines) that includes the optically resolved dendrite like processes. ***B***, Sholl profile of astrocytes in dCA3: no significant change was observed in the arborization of astrocytes in dCA3 with stress. ***C***, ***D***, No significant change was observed in the volume normalized total filament length and total branch points of astrocytes in dCA3 with stress (control: *N* = 11 animals, *n* = 39 cells, stress: *N* = 10 animals, *n* = 32 cells). ***E***, Sholl profile of astrocytes in BA: no significant change was observed in the arborization of astrocytes in BA with stress. ***F***, ***G***, Stress leads to a significant increase in the volume normalized total filament length and total branch points of BA astrocytes (Student's unpaired *t* test, *p < 0.05; control: *N* = 13 animals, *n* = 43 cells, stress: *N* = 11 animals, *n* = 40 cells). Bar insert, Number of animals and number of cells (in parentheses). Scale bar: 20 µm.

The GFAP-positive cell counting and neuropil volume calculation of dye-filled astrocytes were both done in FIJI (NIH). The GFAP-positive cells were identified in a two-channel composite image consisting of the DAPI and GFAP channels and counted manually using the cell counter plug-in. The cell counts were acquired from the dCA3 and BA regions. In the dCA3, cell counts were acquired from a region containing the cell body layer and the molecular layer up to a perpendicular distance of 400 µm. Cell counts are reported as number of GFAP-positive astrocytes per unit volume of brain tissue. For neuropil volume calculation of dye-filled astrocytes, the images were converted to binary format using constant absolute thresholding parameters. The area of non-zero pixels in each optical slice was calculated using the particle analysis plug-in. Using this technique, multiple cross-sectional areas consisting of signals only from the astrocytic processes could be calculated spanning the 3D volume occupied by the cell. The neuropil volume was then calculated by multiplying the sum of areas in all optical slices in an image with the Z-step size, i.e., 0.5 µm (Fig. 2*A*). This method of analysis excludes large, optically resolved spaces occupied by blood vessels, cell bodies and other cellular structures within the territory of the dye-filled astrocytes. Arborization profiles of dye-filled astrocytes and neurons were studied using 3D Sholl analysis in Imaris (Bitplane). Both astrocytes and neurons were traced and 3D rendered as filaments using the *Autopath* algorithm. For astrocytes, a seed point threshold of 0.66 µm (size exclusion criteria for local contrast-based segmentation algorithm) was used to include all the optically resolved, dendrite like processes for 3D reconstruction. For neurons, the same algorithm was used in a semi-automated manner, with manual seed point placements, to ensure complete structural representation and remove seed points for overlapping dendrites from adjoining dye-filled neurons. Using the soma as reference point in the 3D render, the branching profile of individual astrocytes and neurons were analyzed as a function of radial distance from the soma. The Sholl profile for astrocytes is represented as the number of processes intersecting each incremental, concentric Sholl sphere placed 1 µm apart (Fig. 3*B*,*E*). For neurons, the Sholl profile is represented as processes intersecting every concentric Sholl sphere 10 µm apart (Fig. 6*C*).

**Figure 4.**
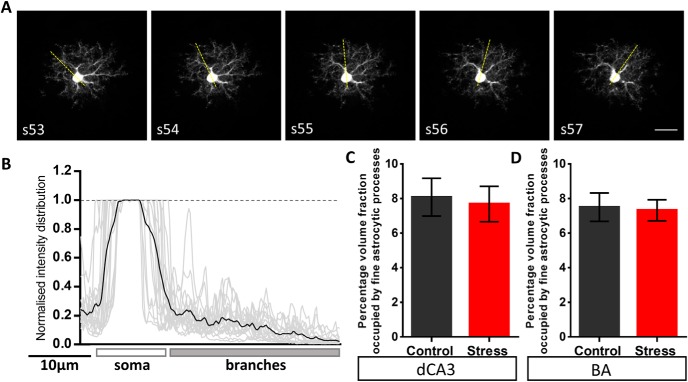
Stress does not affect the volume fraction of fine astrocytic protrusions across the BA and the dCA3 area of the hippocampus. ***A***, Representative background subtracted images of consecutive optical sections (optical section number denoted by the inset text) consisting of the astrocyte soma and its processes. The overlay of yellow dotted line represents the sampling line ROI which was used to perform intensity sampling along the length. The line ROI was rotated at an increment of 20° around the soma (with a random initial angle) throughout the optical sections containing the soma. ***B***, A representative plot of intensity distribution across the length of the sampling ROI. The intensity profile is normalized to the emission signal intensity in the soma. The gray lines represent intensity profiles of individual optical sections containing the soma across multiple astrocytes and the black line represents the mean intensity. ***C***, ***D***, No significant difference was observed in the relative intensity of fine astrocytic processes of astrocytes in the BA or hippocampal dCA3 area (dCA3: control: *n* = 11 cells; stress: *n* = 12 cells, BA: control: *n* = 12 cells; stress: *n* = 15 cells). Scale bar: 20 µm.

The local volume fraction occupied by the fine processes of dye-labeled astrocytes was quantified using a previously published protocol (Medvedev et al., 2014). Briefly, the astrocyte structure was broadly divided into two categories. One category consisted of the astrocytic soma and thick dendrite-like processes and the other consisted of the fine astrocytic protrusions. Using FIJI, the raw image of dye-labeled astrocyte was background subtracted using a rolling ball background subtraction algorithm with a diameter of 30 pixels. The volume fraction occupied by the fine astrocytic protrusions were calculated by sampling emission intensities using a line ROI through the optical sections containing the soma (Fig. 4*A*). The emission intensity profile was normalized to the maximum saturated intensity of the soma (Fig. 4*B*). Next, the normalized intensity peaks from soma and large processes (larger than 1 µm) were excluded by evaluating the base width of the intensity profile using a peak detection algorithm (using MATLAB) based on local minima and maxima. The resulting average intensity values (F_min_) from peaks with base-width < 1 µm represented the volume fraction occupied by the fine astrocytic protrusions.

**Figure 5.**
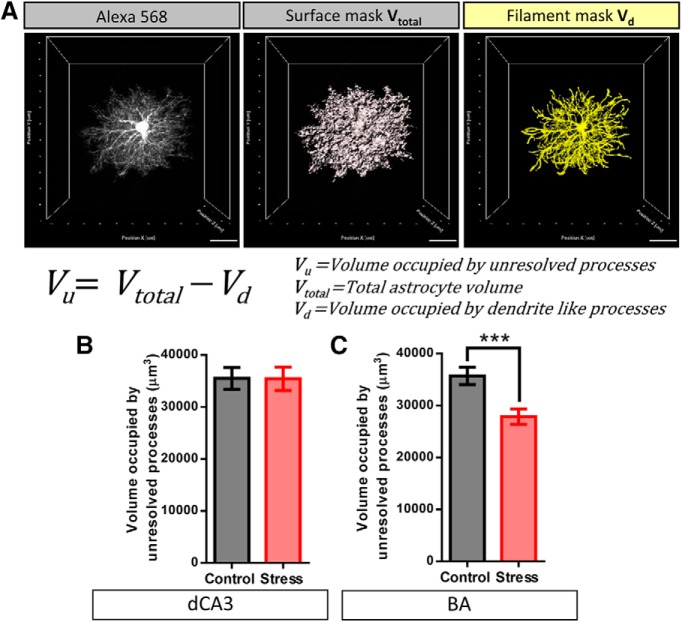
Stress induced reduction in neuropil volume of astrocytes in BA is caused due to a reduction in the overall volume occupied by fine astrocytic protrusions rather than the larger dendrite like processes. ***A***, left, A representative image of Alexa Fluor 568-filled astrocyte. Center, Surface mask depicting entirety of the neuropil volume occupied by the astrocyte (*V_total_*). Right, Filament mask depicting the volume occupied by the optically resolved dendrite like processes (*V_d_*). The volume occupied by the fine (optically unresolved) astrocytic protrusions (*V_u_*) is calculated by subtracting *V_d_* from *V_total_*. ***B***, No significant change was observed in the volume occupied by the fine protrusions of dCA3 astrocytes with stress (control: *n* = 40 cells, Stress: *n* = 32 cells). ***C***, Stress leads to a significant reduction in the volume occupied by fine protrusions of BA astrocytes (Student’s unpaired *t* test, ***p < 0.001; control: *n* = 42 cells, stress: *n* = 38 cells). Scale bar: 20 µm.

The relative contribution of fine astrocytic protrusions to the overall astrocyte territory was assessed by a volume subtraction approach. The volume occupied by the fine astrocyte protrusions (*V_u_*), that lie beyond the diffraction limited resolution of light microscopy, was calculated by subtracting the volume occupied by large dendrite like processes (*V_d_*) from total neuropil volume occupied by astrocyte (*V_total_*; Fig. 5*A*, equation**)**. *V_d_*was calculated by interpolating the dendrite thickness of 3D-reconstructed filaments and the soma. A local intensity threshold algorithm was used that considered the shortest distance from a radial distance map around each seed point to interpolate the dendrite thickness (Fig. 5*A*, third image from the left). *V_total_*was re-calculated from surface masks made in Imaris using the same manual intensity threshold as used for the analysis done in FIJI (Fig. 5*A*, second image from the left).

### Statistical analysis

All statistical analyses were performed in GraphPad Prism (GraphPad Software Inc.). Results are expressed as mean ± SEM. Statistical significance of the effect of chronic stress on GFAP cell numbers were analyzed by Kolmogorov–Smirnov test since the dCA3 dataset was not a normal distribution (confirmed by D’Augostino and Pearson omnibus normality test and Shapiro–Wilk normality test). Statistical significance of the effect of chronic stress on astrocyte neuropil volume, total filament length, total branch points, relative intensity and volume occupied by fine astrocytic protrusions were analyzed by Student’s unpaired *t* test. Comparisons of branching profiles in astrocytes and neurons were done by two-way repeated measure ANOVA followed by *post hoc* Sidak’s multiple comparisons for each segment.

## Results

### Brain region-specific effects of CIS on the number of GFAP-positive astrocytes

Earlier studies have demonstrated that repeated psychosocial stress causes a reduction in the number of astrocytes in the dorsal hippocampus of tree shrews (Czéh et al., 2006). Therefore, we first examined whether 10 d of CIS (2 h/d; Fig. 1*A*) affects the number of GFAP-positive astrocytes in area CA3 of the dorsal hippocampus (dCA3) of adult male Sprague Dawley rats. Chronic stress had no statistically significant effects on the density of GFAP-positive cells in the dCA3 region (number of cells/mm^3^, control: 11,283 ± 222, *n* = 46 fields of view, *N* = 5 animals; stress: 11,325 ± 201, *n* = 55 fields of view, *N* = 6 animals, Kolmogorov–Smirnov test: *p* = 0.2444; Fig. 1*B*,*C*). However, the same stress elicited a significant reduction in the density of GFAP-positive astrocytes in the basal nucleus of the amygdala (BA) in the same brain (number of cells/mm^3^, control: 15,997 ± 594, *n* = 32 fields of view, *N* = 5 animals; stress: 13,669 ± 465, *n* = 34 fields of view, *N* = 6 animals, Kolmogorov–Smirnov test: *p* = 0.0025; Fig. 1*D*,*E*).

### Brain region-specific effects of CIS on neuropil volume occupied by individual astrocytes

Individual astrocytes are known to have highly ramified morphology and occupy non-overlapping physical territories (Bushong et al., 2002). However, all previous observations on stress-induced structural plasticity in astrocytes are based on GFAP expression, which only accounts for a relatively small proportion of the total volume of a single astrocyte and primarily represents the more proximal main shafts of these cells (Bender et al., 2016). Thus, we next focused on the morphology of individual astrocytes by examining the impact of the same 10-d chronic stress on the volume occupied by a single astrocyte. To this end, we quantified the neuropil volume of dye-filled astrocytes in the dCA3 and BA regions (Fig. 2*A*,*B*; Materials and Methods). Stress did not lead to any statistically significant difference in the neuropil volume occupied by dCA3 astrocytes [volume (µm^3^) plotted as animal averages, control: 42,467 ± 2067 µm^3^, *N* = 11 animals, *n* = 45 cells; stress: 42,046 ± 3352 µm^3^, *N* = 10 animals, *n* = 35 cells, Student’s unpaired *t* test: *p* = 0.9143; Fig. 2*C*,*D*]. By contrast, stress resulted in a significant reduction in neuropil volume of astrocytes in the BA [volume (µm^3^) plotted as animal averages, control: 42,606 ± 2172 µm^3^, *N* = 13 animals, *n* = 45 cells; stress: 32,796 ± 2551 µm^3^, *N* = 12 animals, *n* = 44 cells, Student’s unpaired *t* test: *p* = 0.0073; Fig. 2*E*,*F*]. Thus, chronic stress caused significant decreases in both the number of GFAP-positive astrocytes and the neuropil volume of individual astrocytes in the BA, but not the dCA3 area, in the same brain.

### Brain region-specific effects of stress on astrocytic structural remodeling

To further examine stress-induced structural remodeling in astrocytes, we next quantified the arborization profile of large astrocytic ramifications. Sholl analysis was performed on the 3D-rendered thick dendrite-like processes of dCA3 and BA astrocytes. Stress had no significant effects on the arborization pattern (control: *n* = 39 cells; stress: *n* = 32 cells; two-way repeated measures ANOVA: stress factor: *p* = 0.7891; Fig. 3*B*) of dCA3 astrocytes. Volume-normalized total filament length and total branch points of dCA3 astrocytes revealed no significant changes after stress (total filament length/100 µm^3^ and total branch points/100 µm^3^, respectively, plotted as animal averages, control: *N* = 11 animals; stress: *N* = 10 animals, Student’s unpaired *t* test: *p* = 0.2447 and *p* = 0.2962, respectively; Fig. 3*C*,*D*). 3D Sholl analysis of thick dendrite-like processes of BA astrocytes also revealed no significant changes after stress (control: *n* = 43 cells; stress: *n* = 40 cells; two-way repeated measures ANOVA: stress factor: *p* = 0.9283; Fig. 3*E*). However, stress led to a statistically significant increase in both the volume-normalized total filament length and total branch points of BA astrocytes (total filament length/100 µm^3^ and total branch points/100 µm^3^, respectively, plotted as animal averages, control: *N* = 13; stress: *N* = 11, Student’s unpaired *t* test: *p* = 0.0354 and *p* = 0.0383, respectively; Fig. 3*F*,*G*).

### Effects of stress on the volume fraction occupied by fine astrocytic processes

To dissect out the local volume fraction and the relative contribution of thin astrocytic protrusions to the overall neuropil volume occupied by these cells, we divided the astrocyte structure into two broad categories: (1) the soma and thick dendrite-like processes, and (2) the fine astrocytic protrusions which form the astrocyte volume fraction that physically contacts the synapses. Since the fine astrocytic processes do not occupy 100% of the sub-resolution volume, the emission intensity from fine astrocytic processes is proportional to their local volume fraction (Medvedev et al., 2014). The distribution of relative emission intensity from the astrocytic processes showed a gradual reduction towards the periphery of the astrocyte domain owing to higher intensities of the thick dendrite like processes proximal to the soma (signal intensities normalized to the soma; Fig. 4*A*,*B*). First, intensity profile analysis across the line ROI (Materials and Methods; Fig. 4*A*,*B*) revealed that stress had no effect on the percentage volume fraction of fine astrocytic protrusions (F_min_) across the two brain regions [dCA3: control: 8.07 ± 1.09%, *n* = 11 cells; stress: 7.68 ± 1.02%, *n* = 12 cells, Student’s unpaired *t* test: *p* = 0.7954 (Fig. 4*C*); BA: control: 7.49 ± 0.81%, *n* = 12 cells; stress: 7.32 ± 0.61%, *n* = 15 cells, Student’s unpaired *t* test: *p* = 0.8629 (Fig. 4*D*)]. We then used a volume subtraction approach to quantify the overall volumetric contribution of fine astroglial protrusions (*V_u_*) to the total astrocytic territory (*V_total_*; Fig. 5*A*; Materials and Methods). Stress did not elicit a significant effect on either the volume occupied by fine astrocyte protrusions [*V_u_* (µm^3^), control: 35,499 ± 2088, *n* = 39 cells; stress: 35,436 ± 2256, *n* = 32 cells, Student’s unpaired *t* test: *p* = 0.9836; Fig. 5*B*] or the volume occupied by the soma and dendrite-like processes of the dCA3 astrocytes [*V_d_* (µm^3^), control: 6949 ± 406, *n* = 39 cells; stress: 6287 ± 429, *n* = 32 cells, Student’s unpaired *t* test: *p* = 0.2691]. On the other hand, stress resulted in a significant reduction in the volume occupied by fine astrocytic protrusions in the BA [*V_u_* (µm^3^), control: 35,703 ± 1676, *n* = 42 cells; stress: 27,859 ± 1509, *n* = 38 cells, Student’s unpaired *t* test: *p* = 0.0009; Fig. 5*C*] with no significant effect on the volume occupied by the soma and dendrite like processes [*V_d_* (µm^3^), control: 7664 ± 389, *n* = 42 cells; stress: 6796 ± 455.8, *n* = 38 cells, Student’s unpaired *t* test: *p* = 0.1499].

### Effects of the same chronic stress on dendritic morphology of BA principal neurons

**Figure 6.**
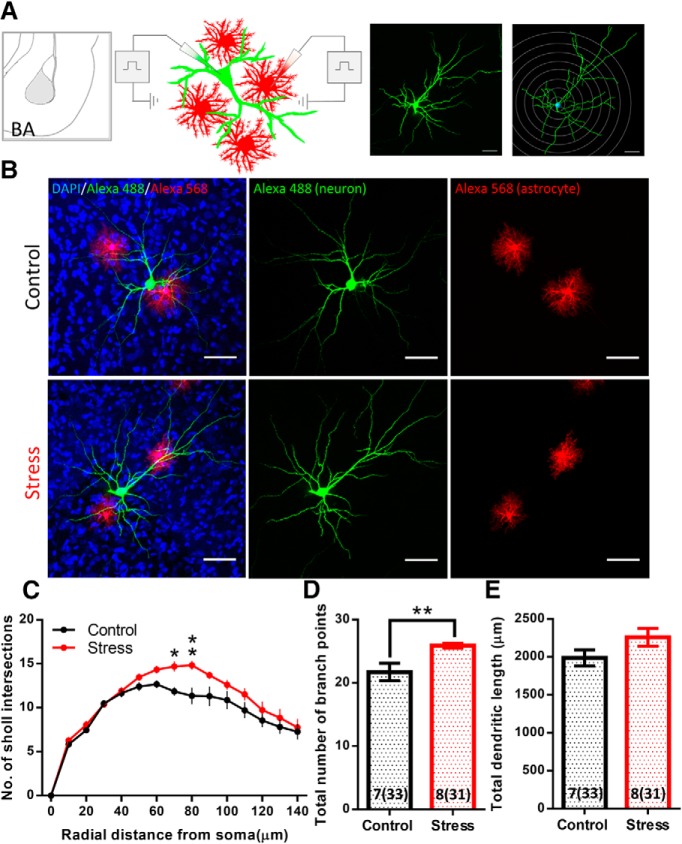
Chronic stress leads to dendritic hypertrophy in the BA principal neurons. ***A***, Schematic of experimental protocol: individual neurons and neighboring astrocytes were filled using Alexa Fluor 488 and Alexa Fluor 568, respectively, by sharp electrode iontophoresis. A confocal image of dye-filled neuron superimposed with concentric Sholl spheres (right). ***B***, Representative confocal image of BA principal neurons (green) with neighboring astrocytes (red). CIS leads to dendritic hypertrophy in the neurons whereas the astrocytes undergo a reduction in their neuropil volume in BA. ***C***, Sholl profile of principal neurons in BA: chronic stress causes significant hypertrophy in the distal branches of BA principal neurons (two-way repeated measures ANOVA: *p* < 0.05, branches between 70 and 80 µm from the soma significantly different in Tukey’s multiple comparison test *p < 0.05; **p < 0.01; control: *N* = 7 animals, *n* = 33 cells, stress: *N* = 8 animals, *n* = 31 cells). ***D***, Chronic stress causes significant increase in the total branch points of principal neurons in BA (Student’s unpaired *t* test, **p < 0.01). ***E***, Chronic stress causes mild increase in the total dendritic length of principal neurons in BA. Bar insert, Number of animals and number of cells (in parentheses). Scale bar: 50 µm.

The results presented so far point to distinct effects of the same stress on astrocytes in the amygdala versus the hippocampus. Growing evidence indicates that stress triggers structural and physiological changes in the amygdalar neurons that are different from those reported earlier in the hippocampus (Chattarji et al., 2015). For instance, the same 10-d CIS, used here, has previously been reported to cause dendritic growth in principal neurons of the basolateral amygdala (Vyas et al., 2002). Hence, in light of the opposite effects of CIS on amygdalar astrocyte number and structure in the present study, we wanted to confirm the efficacy of the same chronic stress in triggering a known morphological effect in amygdalar neurons in the same tissue where we now report these novel effects on astrocytes. Specifically, we wanted to ensure that we also observe the previously reported effects of stress-induced dendritic growth in BA principal neurons. While many of the earlier studies relied on morphometric analyses in Golgi-stained neurons, the present study used dye-filled astrocytes to quantify the morphological effects of stress. Therefore, we next performed morphometric analysis of dye-filled neurons adjacent to the dye-filled astrocytes in the same BA tissue (Fig. 6*A*,*B*). Three-dimensional Sholl analysis revealed that the same chronic stress causes dendritic hypertrophy in BA principal neurons, consistent with earlier reports (Vyas et al., 2002; plotted as animal averages, control: *N* = 7 animals, *n* = 33 cells; stress: *N* = 8 animals, *n* = 31 cells; two-way repeated measures ANOVA: stress factor: *p* = 0.0315; *post hoc* Sidak’s multiple comparison; Fig. 6*C*). The dendritic hypertrophy was also evident in a significant increase in the total number of branch points (plotted as animal averages, control: 22, *N* = 7 animals, *n* = 33 cells; stress: 26, *N* = 8 animals, *n* = 31 cells; Student’s unpaired *t* test: *p* = 0.0087; Fig. 6*D*) and a small, but not significant, increase in total length of dendritic branches [dendritic length (µm), plotted as animal averages, control: 1985 ± 106.6, *N* = 7 animals, *n* = 33 cells; stress: 2258 ± 118.9, *N* = 8 animals, *n* = 31 cells, Student’s unpaired *t* test: *p* = 0.1150; Fig. 6*E*]. Thus, these results confirm that chronic stress in the present study is indeed eliciting a previously established morphological effect, dendritic hypertrophy, in neurons of the BA.

## Discussion

**Figure 7.**
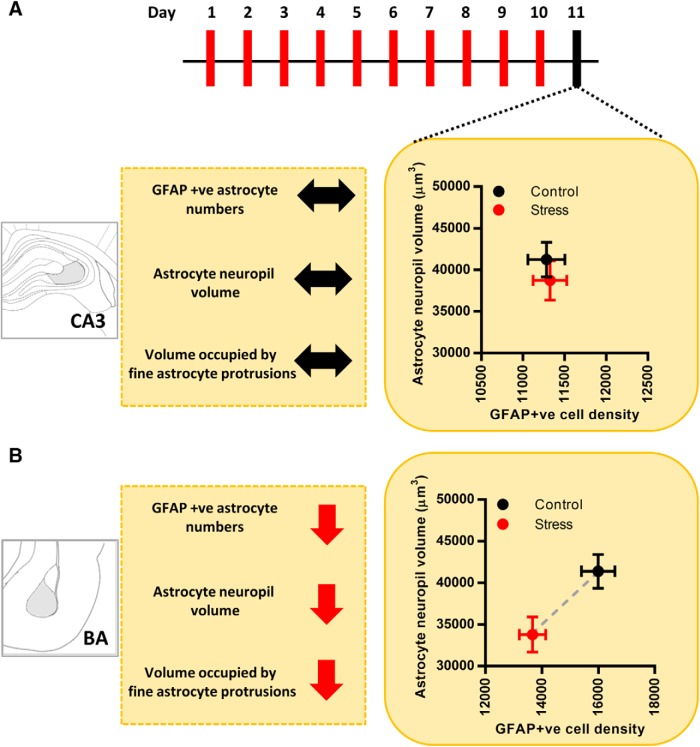
Summary of experimental findings. ***A***, Effects of chronic stress on astrocytes in the dCA3 area. Astrocyte neuropil volume is represented along the *y*-axis and GFAP-positive astrocyte numbers are represented along the *x*-axis. Stress leads to no significant changes in either the neuropil volume or the number of astrocytes in the dCA3 area (control, black; stress, red). Stress leads to no change in the volume occupied by the fine protrusions of dCA3 astrocytes. ***B***, Summarized effects of chronic stress on astrocytes in BA. Astrocyte neuropil volume is represented along the *y*-axis and GFAP-positive astrocyte numbers is represented along the *x*-axis. Stress leads to a significant reduction in both the neuropil volume and the number of astrocytes in BA (control, black; stress, red). Stress also leads to a significant reduction in the volume occupied by the fine protrusions of BA astrocytes; ↔ indicates no change; ↓ indicates decrease.

In this study, we have examined the impact of CIS on astrocytes in the BA and area CA3 of the dorsal hippocampus (dCA3) in rats. We find that this form of repeated stress leads to a reduction in the number of GFAP-expressing astrocytes in the BA. On the other hand, we do not see any effect of the same stress on the number of GFAP-positive astrocytes in the dCA3 area of the same brain (Fig. 7*A*,*B*). The neuropil volume occupied by a single astrocyte, which has highly ramified, spongiform morphology, is an important measure for mapping the physical extent of its association with the neurons (Bernardinelli et al., 2014). Hence, in this study we also conducted a detailed morphometric analysis of dye-filled astrocytes to understand if and how chronic stress induces structural remodeling of these cells. We report, for the first time, that chronic stress causes a reduction in the neuropil volume of BA astrocytes (Fig. 7*B*). No such effect was seen in the dCA3 (Fig. 7*A*). 3D Sholl analysis of optically resolved dendrite-like astrocytic processes in the BA revealed that stress does not affect the overall branching complexity and length of these processes. However, reduction in the overall astrocytic territory leads to a higher packing density of these processes as indicated by the analyses of volume-normalized filament length and branch point (Fig. 3*F*,*G*). This raises the possibility that stress-induced reduction in BA astrocyte territorial volume may be a function of volumetric remodeling at the level of fine astrocytic protrusions. Thus, we used two, indirect but, complementary techniques to gain further insights into the relative contribution of fine astrocytic protrusions to the overall astrocyte territory. Stress did not affect the relative intensity distribution (F_min_), and hence the volume fraction, of fine astrocytic processes across dCA3 and BA (Fig. 4*C*,*D*). However, since the fine astrocytic processes are smaller than the diffraction limited resolution of light microscopy, the uniform average relative intensity across the two brain regions and experimental groups does not rule out the possibility of volumetric reorganization occurring at the level of fine astrocytic processes as reported in an earlier study (Plata et al., 2018). Next, we used the volumetric information from the 3D rendering of dendrite-like processes (*V_d_*) and estimated the relative volume contribution to the astrocyte territory by the fine processes (*V_u_*) using a simple volume subtraction method (Fig. 5*A*). We found that the loss in astrocyte territorial volume in BA is largely due to a reduction in the volume occupied by the fine astroglial protrusions rather than the larger dendrite-like processes (Fig. 5*B*). On the one hand, these indirect measures of the volume occupied by fine astrocytic processes point to the region-specific effects of stress in astrocytic structural re-organization. On the other hand, it also highlights the limitation of such quantification techniques to assess astrocytic leaflets. Since quantification of such structures based on conventional light microscopy techniques is prone to high variability owing to the diffraction-limited resolution and across different labeling methods, it underscores the need to use super-resolution light microscopy or electron microscopy techniques to further assess the nanoscopic volumes occupied by these processes and their interactions with neurons in greater detail (Heller et al., 2017; Scales et al., 2018). Our observations also confirm that the BA principal neurons, in the vicinity of astrocytes that undergo a loss in volume, exhibit dendritic hypertrophy (Fig. 6*C–E*). These observations not only provide new insights into stress-induced structural plasticity of astrocytes but also offer a framework for further analysis of the factors governing the divergent nature of such plasticity across the hippocampus and amygdala.

Previous studies, using both rodent and primate models of stress, have shown that different forms of stress have a significant impact on astrocytes in the hippocampus. For instance, chronic psychosocial stress decreased the number of hippocampal astrocytes in tree shrews (Czéh et al., 2006). Notably, neonatal maternal separation stress has also been shown to cause a reduction in the number of GFAP-positive astrocytes (Musholt et al., 2009). Repeated immobilization stress (6 h/d for 21 d), however, increases GFAP expression in the hippocampus of adult rats (Jang et al., 2008). These divergent findings highlight the need to further examine whether GFAP expression, a widely used metric of changes in astrocytes, by itself is adequate to assess the full morphological impact of stress on hippocampal astrocytes. While earlier studies have reported both decrease and increase in GFAP expression following different forms of stress, we find that the chronic stress paradigm used here has no significant impact on either the morphology or number of dCA3 astrocytes.

Unlike the hippocampus, little is known about the effects of stress on astrocytes in the amygdala. Post-mortem analyses of brain tissue from major depressive disorder (MDD) patients have reported significant reductions in the number of amygdalar astrocytes (Bowley et al., 2002; Altshuler et al., 2010). Our finding on stress-induced decrease in the number of BA astrocytes is consistent with these clinical reports. Astrocytes also exhibit region specific structural plasticity under various physiological, as well as pathological, conditions that have important functional implications for synaptic physiology and overall network function (Bernardinelli et al., 2014). For example, the synaptic coverage by astrocytic processes undergo a significant reduction in the supraoptic nucleus (SON) of lactating rats (Oliet et al., 2001) as well as in the nucleus accumbens (NAc) of cocaine self-administering rats (Testen et al., 2018). Other studies showed the importance of astrocytic synaptic coverage in mediating signaling mechanisms that are involved in maintaining neuronal integrity in the case of peripheral nerve injury (Tyzack et al., 2014) or hippocampal lesions (Wilhelmsson et al., 2006). Hence, our analysis of dye-filled astrocytes also enabled us to extend our morphologic quantifications to the more distal and functionally relevant structural extremities of these cells. This revealed distinct patterns of changes that, as discussed later, may have important functional implications for neurons and their synaptic connections.

The finding that the same chronic stress that is known to elicit contrasting forms of plasticity in neurons of the hippocampus versus amygdala, also has differential effects on astrocytes in these two brain structures, raises several questions that will require further analyses. For instance, are these divergent effects of stress on neurons and astrocytes separate and unrelated processes, or linked to each other? Several findings point to the latter possibility. First, there is growing evidence for a common set of endocrinological and physiologic changes elicited by stress in the hippocampus and amygdala. Among these, elevated levels of glucocorticoids and glutamate appear to be one of the earliest stress-induced changes that are systemic and similar in both brain areas respectively (Lowy et al., 1993; Magariños and McEwen, 1995; Reznikov et al., 2007; Rao et al., 2012; Madan et al., 2018). Yet, the neuronal plasticity mechanisms that ultimately take shape across these two areas reveal strikingly different patterns despite sharing common features in their genesis. Investigations into the underlying mechanisms leading to these contrasting patterns have focused largely on physiological and biochemical processes within neurons. However, astrocytes are also well positioned to mediate some of the initial responses to stress. First, the cellular effects of the adrenal stress hormone corticosterone are not restricted to neurons alone. Corticosterone receptors are also expressed in astrocytes across various brain regions (Wang et al., 2014). Systemic administration of corticosterone in rats reduces GFAP expression in hippocampal and cortical astrocytes (O’Callaghan et al., 1991), which in turn is known to affect astrocytic glutamate reuptake. Further, a study on post-mortem tissue from MDD patients has revealed elevated levels of GR expression in GFAP-positive astrocytes in the amygdala (Wang et al., 2014). These studies point to a pivotal role played by stress-induced increase in corticosterone levels in triggering structural reorganization of astrocytes, as well as astrocytic glutamate reuptake. Second, not only do the astrocytes form physical barriers against glutamate spillover from synapses, they also form the major glutamate reuptake machinery in the brain (Araque et al., 1999). Altered expression and activity of the glutamate transporters present on astrocytes, GLT-1 and GLAST, has been implicated as a key factor underlying the pathophysiology of several neural disorders (Anderson and Swanson, 2000). Third, a growing body of research indicates a bidirectional relationship between neuronal activity and astrocyte structural plasticity in health and disease. Rapid structural rearrangements of the fine astrocytic processes occur during the induction of hippocampal long-term potentiation (LTP), a well-studied cellular substrate for learning and memory (Lushnikova et al., 2009; Perez-Alvarez et al., 2014). Astrocyte coverage of synapses in the amygdala also undergo a striking reduction during Pavlovian fear conditioning (Ostroff et al., 2014). Multiple lines of evidence have also highlighted the importance of cross talk between astrocytes and neurons in the pathophysiology of multiple disease conditions. For instance, distal branches of astrocytes undergo atrophy along with gliogenesis in the hippocampal CA1 region after status epilepticus which, in turn, correlates with the neurodegeneration observed in epilepsy (Plata et al., 2018). Other studies have implicated cellular and molecular alterations in astrocytes in factors contributing to the progression of Alzheimer’s disease (Binder and Steinhäuser, 2006). Although we have only identified differential effects of stress on astrocyte number and morphology in the hippocampus versus amygdala, these changes may be indicative of important functional differences as well. Specifically, future studies will be required to examine if the brain region-specific contrasting effects of stress are caused by differences in glutamate reuptake by astrocytes in these two areas. For example, together with earlier reports of reduced spine-density on dorsal hippocampal pyramidal neurons (Pawlak et al., 2005), the absence of any stress-induced change in astrocyte neuropil volume or volume occupied by fine protrusions reported here, may result in synaptic coverage by hippocampal astrocytes remaining either unchanged or being enhanced following repeated stress. By contrast, reduction in astrocyte numbers, neuropil volume and volume occupied by the fine protrusions of amygdalar astrocytes reported here, may cause chronic stress to decrease astrocyte coverage in the BA. Since stress also increases dendritic arborization and spine density in principal neurons of the BA, this may result in a higher number of synapses being exposed due to loss of adequate coverage by astrocytic processes. This, in turn, may result in extra-synaptic spillover of glutamate due to insufficient reuptake of high levels of glutamate from the synapse. This could affect how stress-induced increase in glutamate levels are regulated in the amygdala, thereby triggering synaptic changes that eventually culminate in differential effects compared to those seen in the hippocampus. Interestingly, in a recent study, a single 2-h episode of the same immobilization stress triggered an immediate upregulation of protein synthesis in neurons, as well as astrocytes, in both the dCA3 area and BA. However, these two areas eventually exhibited opposite temporal profiles of protein expression 10 d after the end of stress (Madan et al., 2018).

In conclusion, a majority of past studies have focused on neuronal, cell autonomous effects of stress in the brain. Results presented here highlight the need to extend future research to examine non-cell autonomous astroglial-mediated effects elicited by stress, and how these vary with brain regions. Thus, our findings, along with earlier research, implicate a potentially important and yet understudied role for glia in stress-induced plasticity.

## Synthesis

Reviewing Editor: Alfonso Araque, University of Minnesota Decisions are customarily a result of the Reviewing Editor and the peer reviewers coming together and discussing their recommendations until a consensus is reached. When revisions are invited, a fact-based synthesis statement explaining their decision and outlining what is needed to prepare a revision will be listed below. The following reviewer(s) agreed to reveal their identity: Michael Scofield, Alexey Semyanov.

The reviewers have found merit and interest on the findings reported. Reviewer # 1 is largely satisfied with the new analysis and authors´ responses. However, there are still some concerns that need to be addressed (see detailed comments below). Reviewer #2 is largely unsatisfied with the responses provided. Besides addressing the specific comments detailed below, the following general issues, in which we agreed by consensus after a consultation discussion, are required to be careful considered:

1) Authors should consider that astrocytic processes do not occupy 100% of the space within the astrocytic domain. The provided values may represent an underestimation. Although it may not preclude the major conclusion of the manuscript, this is an important issue that needs to be properly addressed. I concur with reviewer´s suggestion of measuring astrocyte VF as in Medvedev et al, 2014. If there is any compelling reason to prevent it, I would suggest you provide a reasonable explanation.

2) Investigating the link between astrocyte atrophy and neuronal hypertrophy would improve the manuscript. However, we understand that this is a major challenge that probably is out of the scope of the present manuscript. We found unusual, but finally acceptable, removing some of this data, which may be controversial. If no further exploration of the topic is provided, authors should diminish the length and strength devoted to the issue in the Abstract.

In summary, the reviewers and myself agree that the manuscript reports and interesting finding that is worth to be reported to the neuroscience community. However, important aspects of the study need to be carefully considered to provide solid results supporting the important conclusion.

I urge you to fully address these general comments and the specific reviewers´ concerns, which may require less than 2 months' work, to proceed further.

Specific comments of the reviewers:

Reviewer 1.

The paper provides an interesting methodology for the examination perisynaptic processes of astrocytes. Scientifically, an interestingly dichotomous relationship between astrocyte and neuron shape is presented.

Specific comments

Values used for astrocyte filament analysis, like total filament length, should be normalized to each individual cells surface mask, what the authors deem Vtotal. This would give readouts in filament length/cubic micron. This removes some variability and may help the authors data. Once this normalization is done on a cell by cell basis then animal averages can be constructed.

The same should be done and shown with the sum of sholl intersections. The authors show each value but a single total value can be obtained for each astrocyte, which can then be normalized to Vtotal as described above. Normalizing these values for each cell is crucial as the authors report a difference in astrocyte volume. Moreover these data be informative for interpreting figures 3C and 3F.

As the authors have added significant amounts of data, the number of cells and the number of animals used should be depicted within the bar graphs of each figure.

The estimation of the volume of unresolved perisynaptic astrocyte processes or PAPs is interesting and in line with the other data presented, but intrinsically would be difficult to replicate as the extent of unresolved signal would differ from imaging system to imaging system and vastly across different labels and experimental designs. Moreover, it appears shrinking of unresolved processes is largely an artifact of the decreased overall volume and decreased structural complexity of astrocytes shown in 2F and 3E/F respectively. I think it is an interesting part of the story but some discussion of these caveats should be added.

No description of how Z-step size was selected, or the other optical parameters that would potentially contribute to over or under sampling, is made. The authors should discuss if cells were appropriately sampled according to the nyquist theorem.

The authors should consider sighting other astrocyte imaging papers from the Scofield and Reissner groups that investigate how astrocyte structure and synaptic contact may relate to glutamate spillover / overflow.

https://www.ncbi.nlm.nih.gov/pubmed/26946381

https://www.ncbi.nlm.nih.gov/pubmed/30147645

Reviewer 2.

The authors have made an attempt to address some of my comments, however mostly unsatisfactorily. Overall, the manuscript has not been significantly improved, if not worsened.

1. I suggested to perform Sholl analysis on astrocytes and measure the volume fraction of unresolved astrocytic processes. Indeed, Sholl analysis was performed. However, the authors did not get the meaning of astrocytic volume fraction. Volume fraction (VF) is the proportion of the space occupied by astrocytic processes. Astrocytic processes do not occupy 100% of the space within the astrocyte domain, leaving space for the other structures. Medvedev et al suggested a method to estimate VF of the processes which are beyond the optical resolution. They compared their fluorescence to the fluorescence of the soma, assuming that the soma occupies 100% of the space. Here the authors performed absolutely unrelated procedures and called it “adapted from a previously reported protocol”. This is rather misleading for the reader.

2. I suggested strengthening the link between neuronal hypertrophy and astrocyte atrophy suggested in the original manuscript. The authors answered that they actually did not imply such a link and removed some of the original data. Nevertheless, they spend 1/3 of the abstract to discuss the different effects of stress on neurons and astrocytes. Such an answer is not satisfactory. I think the link between changes in neurons and astrocytes need to be explored. This would strengthen the manuscript.

3. The discussion of astrocyte remodeling is insufficient. The astrocyte remodeling occurs at the level of both fine astrocytic processes and astrocytic branches. For example, transient changes in perisynaptic astrocytic processes occur after LTP. Remodeling of astrocytic branches occurs in aging, in Alzheimer's disease, after status epilepticus etc.

Minor comments

1. Page 1 line 14 “Finally, we confirmed previous reports that chronic stress triggers dendritic growth in dye-filled BA principal neurons.” Why is it finally? Now the authors say that the tests on the neurons were done merely to confirm the effect of stress.

2. In the revised manuscript the means are given for animals throughout the text. There is no need to state the number of cells. Otherwise, it looks confusing and gives the impression that means are presented for individual cells.

3. The authors used a t-test for figure 1C. However, the figure shows that the median does not match the mean (+). This suggests that this is not a normal distribution and the t-test cannot be used.
